# Administration of plasma-derived coagulation factor VIII during the perioperative period of mastectomy for breast cancer with acquired von Willebrand syndrome

**DOI:** 10.1186/s40792-018-0528-y

**Published:** 2018-09-17

**Authors:** Ritsuko Sasaki, Yoshiya Horimoto, Ju Mizuno, Yoko Edahiro, Tsukasa Ohmori, Norio Komatsu, Mitsue Saito

**Affiliations:** 1 Department of Breast Oncology, Juntendo University School of Medicine, 2-1-1 Hongo, Bunkyo-ku, Tokyo, 113-0033 Japan; 2 Department of Anesthesiology and Pain Medicine, Juntendo University School of Medicine, 2-1-1 Hongo, Bunkyo-ku, Tokyo, 113-0033 Japan; 3 Department of Hematology, Juntendo University School of Medicine, 2-1-1 Hongo, Bunkyo-ku, Tokyo, 113-0033 Japan; 4 Department of Biochemistry, Jichi Medical University School of Medicine, Tochigi, 329-0498 Japan

**Keywords:** von Willebrand syndrome, Breast cancer, Perioperative management, Polycythemia vera

## Abstract

**Background:**

Acquired von Willebrand syndrome (aVWS) is a rare bleeding disorder with laboratory findings similar to those of congenital von Willebrand disease (VWD). Patients with aVWS may require prophylactic treatment to prevent excessive bleeding following surgery. To our knowledge, to date, there have been no reports on perioperative management for breast cancer patients with aVWS.

**Case presentation:**

A 60-year-old woman with breast cancer was diagnosed with aVWS due to polycythemia vera. Pre-operative laboratory testing showed a high platelet count and low von Willebrand factor (VWF) activity. The VWF activity did not improve despite an attempt to suppress platelet count with hydroxyurea. Therefore, we decided to perioperatively supplement with plasma-derived factor VIII (FVIII) containing von Willebrand factor (FVIII/VWF concentrates) to perform curative surgery for breast cancer safely. Consequently, the patient did not develop hemorrhage during/after surgery and was discharged on postoperative day 7, as planned, without problems.

**Conclusions:**

For a patient with aVWS, which carries a high risk of hemorrhage during the perioperative period, initiation of appropriate management like supplementation of FVIII/VWF concentrates might enable safe curative surgery for breast cancer, and collaboration with the hematology department is critical.

## Background

### Outline of von Willebrand syndrome

Von Willebrand factor (VWF) is a crucial factor for primary hemostasis after vascular damage as it functions in platelet adhesion. Since VWF and coagulation factor VIII (FVIII) circulate in blood as a tight complex, VWF stabilizes the structure of FVIII and protects from proteolytic degradation [1]. Either a numerical disorder or dysfunction of VWF could cause hemorrhagic diathesis [2]. Von Willebrand disease (VWD) is defined as congenital if a patient has abnormality of the *VWF* gene, or acquired if the disease developed following malignant hematologic disease or cardiovascular disease [3, 4]. Acquired von Willebrand syndrome (aVWS) is considered rare, though the exact prevalence remains unknown. As far as we know, only 266 cases were reported from 1968 to 2000, the first of which was reported by Simone et al. [3–7]. A recent report suggested that the incidence of aVWS has increased in patients with cardiovascular disease such as aortic stenosis [8, 9]. One reason for this phenomenon may be the rise in awareness of this disease among cardiologists.

### Perioperative management for patients with aVWS

Supplementation of FVIII containing von Willebrand factor (FVIII/VWF concentrates) and desmopressin, which promotes the release of VWF, have reportedly been employed for patients with VWD as part of perioperative management. FVIII/VWF concentrates might be used for several consecutive days after surgery to maintain effective levels in the blood [3, 10]. Tight blood monitoring during the perioperative period is advisable, particularly for a patient undergoing major cardiac or orthopedic surgery [10–13].

We herein report a breast cancer patient with aVWS at high risk of hemorrhage during the perioperative period. The patient safely underwent curative surgery for her breast cancer with supplementation of FVIII/VWF concentrates. To our knowledge, to date, there have been no reports on perioperative management for breast cancer patients with VWF; thus, we believe that our report may provide useful information for clinicians involved in breast cancer treatments or other invasive procedures.

## Case presentation

### 60-year-old female

A diagnosis of polycythemia vera (PV) with *JAK2* V617F mutation was made at age 47, and aspirin therapy was initiated. However, the treatment was changed to and continued with hydroxyurea (HU) and phlebotomy due to persistent purpura. At age 56, calcifications of the right breast were noted during mammography screening. During stereotactic vacuum-assisted biopsy, she experienced difficult hemostasis and necrotic ulcer of the skin developed due to delayed wound healing from hematoma. The biopsy of the breast tissue showed sclerosing adenosis, and the patient was followed up routinely.

At age 60, ultrasound from a routine check-up showed a hypoechoic lesion in the upper-inner area of the left breast. Pre-biopsy laboratory testing showed a high platelet count of 688,000/μL and low VWF activity of 28% (reference value 60–170%); thus, the patient was diagnosed with aVWS. Low VWF activity is usually accompanied by a tendency for bleeding, but unlike congenital VWD, VWF activity may be normalized with treatment of the underlying condition (3). Therefore, we attempted to increase the HU dose, but there was no improvement and the biopsy was performed with administration of FVIII/VWF concentrates. The biopsy result showed apocrine carcinoma, so the patient was referred to our hospital for treatment.

**Fig. 1:**
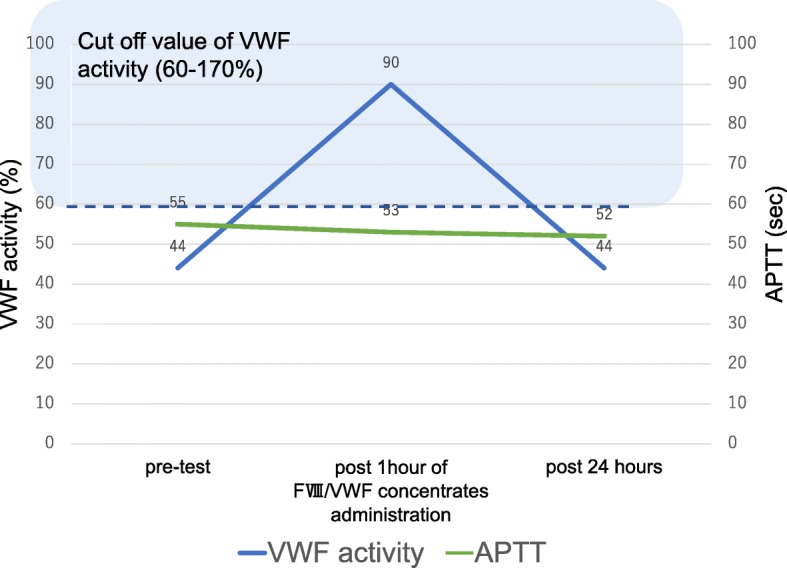
Change in VWF activity and APTT after test administration of FVIII/VWF concentrates. VWF activity is higher than the reference values at 1 h post-administration VWF: von Willebrand factor, APTT: activated partial thromboplastin time,

Although treatment for PV was continued, prolonged activated partial thromboplastin time (APTT), low VWF activity, and low activity of factors IX, XI, and XII persisted. Two units of fresh frozen plasma (FFP) were used to replete coagulation factors, which did not result in improvement in APTT and a mixing study showed an inhibitor pattern, plus the patient was positive for lupus anticoagulant (LA), so FFP alone was determined to be inadequate. FVIII/VWF concentrate administration was tried instead in an outpatient setting, which yielded VWF/VIII activity higher than the reference values at 1 h post-administration (Fig. 1), so we determined that mastectomy was possible with FVIII/VWF concentrates. Despite the use of FVIII/VWF concentrates, APTT did not improve, due to not only the presence of aVWS, but also LA positivity.

**Fig. 2:**
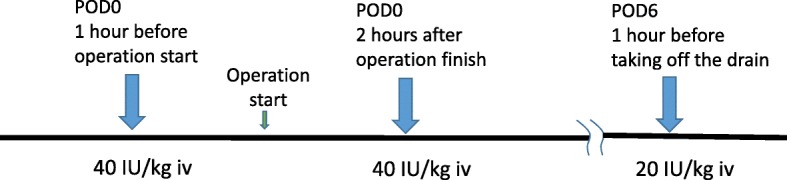
Timeline of the perioperative management for administration of FVIII/VWF concentrates. IU, international unit; iv, intravenous dosage; POD, postoperative day

Perioperative management was done in close collaboration with the hematology, anesthesiology, and pathology departments according to the timeline on administration of FVIII/VWF concentrates shown in Fig. 2. The dosage of FVIII/VWF concentrates was planned according to Table 1. Table 1 shows the initial dosing recommendations for FVIII/VWF concentrate replacement for the prevention of bleeding in perioperative coagulation management [10, 14]. Left mastectomy and sentinel lymph node biopsy were performed with an operative duration of 63 min and intraoperative blood loss of 3 mL. The drain was placed only above the inframammary fold; a shorter drain was used to reduce stimulation during its removal. Suture ligation was used rather than an electric scalpel for better hemostasis. To ensure a short operative duration, the pathology department was informed in advance so that lymph node biopsy could be quickly diagnosed.

**Table 1:** Perioperative coagulation management on administration of FVIII/VWF concentrates in patients with aVWS

		Therapeutic goal VWF activity	Loading dose	Maintenance dose	Number of doses a day
Minor surgery	Day of surgery	50–100%	40–60 IU/kg	30–40 IU/kg	2
	2–7 days	30–50%		20–30 IU/kg	1–2
Major surgery	Day of surgery to 3 days	100%	30–60 IU/kg	40 IU/kg	2–3
	4–7 days	50–100%		30–40 IU/kg	2
	8–14 days	30–50%		20–30 IU/kg	1–2

*IU* international unit

FVIII/VWF concentrates were used again at the time of drain removal on postoperative day 6. No bleeding was observed, and the patient was discharged. The surgical site healed without major complications, and no bleeding was observed during outpatient seroma drainage.

The pathological diagnosis result of the surgical specimen showed a 5-mm-diameter apocrine carcinoma with NG2 ly (−), v (−), ER (−), PgR (−), HER2 (1+), Ki-67 (10%), pT1aN0M0 Stage I. Because the patient had a triple-negative pT1aN0 tumor, adjuvant chemotherapy was not performed, and further postoperative follow-ups are planned.

### Discussion

It is reported that one in ten patients with PV may develop aVWS, and most of such patients develop bleeding disorders [15]. In the present case, VWF activity did not improve despite the attempt to suppress platelet count with HU. FVIII/VWF concentrates were used during perioperative management instead, as it was effective in improving VWF/FVIII activity. Meanwhile, perioperative use of desmopressin is not recommended for major surgery, as repeated use can lead to tachyphylaxis [16, 17]. The reference dose of FVIII/VWF concentrates is reported for various invasive procedures [10], but there is no clear standard. Administration is between one and three times per day, and use is recommended when the patient shows a tendency for bleeding. High plasma levels of FVIII are thought to be correlated with the risk of thrombosis [18–20]. It should especially be used with caution in patients who have known risk factors for venous thrombosis such as old age, previous thrombosis, obesity, and cancer [13]. One report stated that levels of circulating FVIII greater than 150 IU/dL yielded a high risk of thrombosis with an adjusted odds ratio of 4.8 (95% CI 2.3–10.0) [21].

Test administration of FVIII/VWF concentrates was done with the confirmation of VWF/FVIII activity higher than the reference values at 1 h post-administration. Dosage times were scheduled based on the drug effect duration. Since VWF levels require 2–5 days to measure, FVIII level and APTT are useful for the timely evaluation of a patient’s coagulation status. However, if prolonged APTT is related to another pathophysiology as in this case, it is useful to measure FVIII/VWF activity after a trial dose of FVIII/VWF concentrates.

As for systemic therapies, there are some drugs that might cause hemorrhage such as cyclophosphamide and bevacizumab. However, to our knowledge, there is no established evidence linking patients with aVWD to increased risk of adverse events during systemic therapies. Thus, we believe that control of background disease and decent monitoring of FVIII level are crucial if systemic treatments are needed.

## Conclusions

For a patient with aVWS, which carries a high risk of hemorrhage during the perioperative period, initiation of appropriate management like supplementation of FVIII/VWF concentrates might enable safe curative surgery for breast cancer, and collaboration with the hematology department is critical.

## Abbreviations

APTT: Activated partial thromboplastin time
aVWS: Acquired von Willebrand syndrome
FFP: Fresh frozen plasma
HU: Hydroxyurea
LA: Lupus anticoagulant
PV: Polycythemia vera
VWD: Von Willebrand disease
VWF: Von Willebrand factor

## References

[1] Lenting PJ, CJ VANS, Denis CV (2007). Clearance mechanisms of von Willebrand factor and factor VIII. J Thromb Haemost.

[2] Rodeghiero F, Castaman G, Dini E (1987). Epidemiological investigation of the prevalence of von Willebrand’s disease. Blood.

[3] Tiede A, Rand JH, Budde U, Ganser A, Federici AB (2011). How I treat the acquired von Willebrand syndrome. Blood.

[4] Federici AB, Rand JH, Bucciarelli P, Budde U, van Genderen PJ, Mohri H (2000). Acquired von Willebrand syndrome: data from an international registry. Thromb Haemost.

[5] Simone JV, Cornet JA, Abildgaard CF (1968). Acquired von Willebrand’s syndrome in systemic lupus erythematosus. Blood.

[6] Tefferi A, Nichols WL (1997). Acquired von Willebrand disease: concise review of occurrence, diagnosis, pathogenesis, and treatment. Am J Med.

[7] Nitu-Whalley IC, Lee CA (1999). Acquired von Willebrand syndrome--report of 10 cases and review of the literature. Haemophilia.

[8] Sedaghat A, Kulka H, Sinning JM, Falkenberg N, Driesen J, Preisler B (2017). Transcatheter aortic valve implantation leads to a restoration of von Willebrand factor (VWF) abnormalities in patients with severe aortic stenosis - incidence and relevance of clinical and subclinical VWF dysfunction in patients undergoing transfemoral TAVI. Thromb Res.

[9] Saad RA, Lwaleed BA, Kazmi RS (2013). Gastrointestinal bleeding and aortic stenosis (Heyde syndrome): the role of aortic valve replacement. J Card Surg.

[10] Miesbach W, Berntorp E (2017). Von Willebrand disease - the 'Dos' and 'Don'ts' in surgery. Eur J Haematol.

[11] Franchini M (2008). Surgical prophylaxis in von Willebrand's disease: a difficult balance to manage. Blood Transfus.

[12] Baldeo CM, Rivera CE, Tun HW, Vishnu P (2018). Pharmacokinetics-based clinical management of acquired von Willebrand syndrome: a case report. J Blood Med.

[13] Lethagen S, Kyrle PA, Castaman G, Haertel S, Mannucci PM (2007). von Willebrand factor/factor VIII concentrate (Haemate P) dosing based on pharmacokinetics: a prospective multicenter trial in elective surgery. J Thromb Haemost.

[14] Federici AB (2005). Management of von Willebrand disease with factor VIII/von Willebrand factor concentrates: results from current studies and surveys. Blood Coagul Fibrinolysis.

[15] Mital A, Prejzner W, Swiatkowska-Stodulska R, Hellmann A (2015). Factors predisposing to acquired von Willebrand syndrome during the course of polycythemia vera - retrospective analysis of 142 consecutive cases. Thromb Res.

[16] Rodeghiero F, Castaman G, Mannucci PM (1991). Clinical indications for desmopressin (DDAVP) in congenital and acquired von Willebrand disease. Blood Rev.

[17] Ozgonenel B, Rajpurkar M, Lusher JM (2007). How do you treat bleeding disorders with desmopressin?. Postgrad Med J.

[18] Jenkins PV, Rawley O, Smith OP, O’Donnell JS (2012). Elevated factor VIII levels and risk of venous thrombosis. Br J Haematol.

[19] Kraaijenhagen RA, in’t Anker PS, Koopman MM, Reitsma PH, Prins MH, van den Ende A (2000). High plasma concentration of factor VIIIc is a major risk factor for venous thromboembolism. Thromb Haemost.

[20] Miesbach W, Krekeler S, Wolf Z, Seifried E (2015). Clinical use of Haemate(R) P in von Willebrand disease: a 25-year retrospective observational study. Thromb Res.

[21] Koster T, Blann AD, Briet E, Vandenbroucke JP, Rosendaal FR (1995). Role of clotting factor VIII in effect of von Willebrand factor on occurrence of deep-vein thrombosis. Lancet (London, England).

